# Effect of Food Additive Citric Acid on The Growth of Human Esophageal Carcinoma Cell Line EC109

**DOI:** 10.22074/cellj.2016.4716

**Published:** 2016-09-26

**Authors:** Xiaoguang Chen, Qiongxia Lv, Yumei Liu, Wen Deng

**Affiliations:** Animal Science and Technology School, Henan University of Science and Technology, Luoyang, China

**Keywords:** Citric Acid, Cytotoxicity, Esophageal Carcinoma Cell EC109, Cell Proliferation, Apoptosis

## Abstract

**Objective:**

Today, esophageal cancer (EC) has become one of the most common cancer types in China. Therefore, new drug and therapeutic strategies are urgently needed to improve postoperative survival rate of patients with EC. As a food additive, several lines of evidence have shown that citric acid can be served as glycolysis suppressor to inhibit growth of some tumor cells. However, little is known about the effect of this organic acid on the growth of human esophageal carcinoma cell line, EC109.

**Materials and Methods:**

In this experimental study, cell proliferation rate was determined using MTT assay. Apoptotic morphological changes were evaluated by fluorescent microscopy using Hoechst 33258 staining. Cell apoptosis rate and mitochondrial membrane potential (MMP) were detected using flow-cytometry. Effect of citric acid on cellular membrane permeability was assessed by measuring lactate dehydrogenase (LDH) activity, using LDH assay kit.

**Results:**

Compared to the control group, there was a marked decrease in cells proliferation when the cells were treated with higher citric acid concentrations (800, 1600 μg/ml). Typical apoptotic morphology of EC109 cells was observed upon treatment with citric acid, such as chromatin condensation and appearance of apoptotic body. Cell apoptotic indexes were significantly increased (P<0.01) after treatment with citric acid at the concentration of 400-1600 μg/ml. Extracellular LDH activity and loss of MMP in all of the treated groups were significantly higher than control (P<0.05), in a dose-dependent manner.

**Conclusion:**

These results suggest that citric acid prevents EC109 cell growth by inhibiting cell proliferation and inducing apoptosis, which perhaps offers some theoretical guidance for its application in EC treatment.

## Introduction

As a malignant tumor, esophageal carcinoma (EC) is the eighth most common type of malignancy and the sixth most common leading cause of cancer-related death around the world. Particularly in China, according to the annual report on estimation of cancer status, crude mortality rate of EC accounts for about 12% of all cancer deaths and place on the second rank of cancer related death, after gastric cancer. In the past few decades, even though the mortality of EC had been sharply decreased with development of diagnosis and treatment techniques, mortality rate is still very high with only a five-year survival rate of 30% EC patients (1). At present, China still suffers a great disease burden from EC (2). Therefore, to improve postoperative survival chance in EC patients, there is an urgent need to find novel agents effectively treating this disease. Now, several lines of evidence have clearly indicated that metabolic properties of cancer cells are very different from those of normal cells. In detail, healthy cells mainly rely on oxidative phosphorylation, whereas cancer cells are more inclined to employ aerobic glycolysis for proliferation. Even in the presence of oxygen, cancer cells are more dependent on aerobic glycolysis, rather than on oxidative phosphorylation (3). In view of glycolytic phenotype of cancer cells, targeting metabolic dependence could perhaps be considered as an alternative approach to repress growth and metastasis of EC cells (4). 

Citric acid (CA) is a naturally organic acid presented primarily in juice of some fruits and vegetables, especially in citrus fruits (5). Relying on its superior physicochemical properties, this organic acid is now widely used as a food additive/preservative in many processed foods, an ingredient in cosmetic products, a powerful cleaning agent and so on. In the biochemical process, CA is the main chemical intermediate of tricarboxylic acid cycle (TCA) which is the final common oxidative pathway for carbohydrates, fats and amino acids, involved in the chemical conversion of carbohydrates, fats and proteins into carbon dioxide and water to generate energy (6). Therefore, TCA cycle is the most important central pathway connecting almost all of the individual metabolic pathways. Meanwhile, CA level directly influences the TCA cycle activity. As the main route of energy production available for the cells, glycolysis is a fairly complex process involving many enzymes to control a series of biochemical reactions for glycolytic breakdown of glucose, such as phosphofructokinase (PFK), pyruvate kinase (PK) and lactate dehydrogenase 1 (LDH1). As the rate-determining enzyme in glycolysis, PFK catalyzes the first committed step of glycolysis and thus represents an essential metabolic control point for carbohydrate utilization. PK, the last rate-limiting enzyme of glycolysis, catalyzes the conversion of phosphoenolpyruvate and ADP into pyruvate and ATP. Studies showed that PK is predominantly expressed in tumor cells, which is necessary for aerobic glycolysis and provides the growth advantage to tumors (7,8). For instance, Huang et al. (9) showed that in the presence of HSP40, a novel binding partner of PK, level of the latter enzyme was down-regulated and glycolysis was inhibited, which further blocked growth of cancer cells. LDH1 regulates final step of glycolysis that converts pyruvate to generate lactate, as an essential factor for the survival of most tumor cells (10). For example, findings of Zhao et al. (11) showed that knockdown of LDH1 in tumor cells decreased ability of cell proliferation under hypoxic conditions, and thus repressed tumorigenicity. Accordingly, suppression of the above enzyme activities by inhibitors can hinder growth of tumor cells via impairment of anaerobic glycolysis. Among the inhibitors, CA is now considered as one of the effective agents suppressing these glycolytic enzyme activities (4). As reported by Jenkins et al. (12), citrate is a potent natural inhibitor of glycolysis functioning through blockade of PFK activity. Data obtained by Hatzivassiliou et al. (13) and Li et al. (14) suggested that SB-204990 (the chemical inhibitor for ATP citrate lyase) could increase citrate level, which in turn inhibited aerobic glycolysis, thereby producing anti-cancer effects. The study of Liu et al. (15) demonstrated that addition of sodium citrate remarkably reduced activities of PFK and PK, and subsequently attenuated glycolytic flux. Findings of Gu and Zhao (16) showed that citrate greatly exhibited inhibitory effect on LDH1, indicating the regulatory effect of this organic acid on the last step of glycolysis. 

Considering that CA acts as a glycolytic inhibitor and negatively impact on tumor growth, in this study, biochemical methods and cell biological techniques were employed to explore the effect of CA on proliferation and apoptosis of EC109 cells after administrating different doses of CA, which may provide a new therapeutic idea for human EC therapy. 

## Materials and Methods

CA used in this experiment was of the analytical grade and obtained from ChengDu Kelong Chemical Reagent Company (ChengDu, China). Dulbecco’s Modified Eagle’s Medium (DMEM) was purchased from Hyclone (Logan, USA). Hoechst Staining Kit, Annexin V-FITC/PI Apoptosis Detection Kit, JC-1 Mitochondrial Membrane Potential Assay Kit and cell culture reagents were purchased from Beyotime Institute of Biotechnology (Hangzhou, China). MTT Cell Proliferation Assay Kit and LDH Assay Kit were respectively supplied by Beijing Dingguo Biotech Co., Ltd. (Beijing, China) and NanJing JianCheng Bioengineering Institute (Nanjing, China). 

### Cell line and culture conditions

Human EC cell line used in this experimental study, EC109, was procured from Research Center of Laboratory Animals, Fourth Military Medical University (Xian, China). This study has been approved by Ethical Committee at Henan University of Science and Technology (China). EC109 cells were maintained in DMEM medium supplemented with 10% (v/v) fetal bovine serum (FBS), 100 U/ml penicillin and 100 μg/ml streptomycin (Gibco Laboratories, South America Invitrogen). Cells were grown at 37˚C in a humidified atmosphere containing 5% CO_2_ . Once the cells reached ~90% confluency, they were detached using 0.05% trypsin (Wuhan Boster Biological Technology Ltd., China) and sub-cultured at a 1:2 split ratio. The cells used in this study were required to stay in log phase. 

### Grouping and citric acid treatment

At 40-50% confluency (about 48 hours post seeding), the cultivated cells were randomly assigned to four groups with five replications per group, including the control group (0 μg/ml CA), low-dose CA group (400 μg/ml), middle-dose CA group (800 μg/ml) and high-dose CA group (1600 μg/ml). The three latest groups were treated with different concentrations of CA; while control group was treated with equivoluminal serum-free-medium, instead of CA. The cells were pooled for use after 48 hours of CA treatment in CO_2_ incubator. 

### Cell proliferation assay

The effect of CA on cell viability was evaluated using MTT method. For this purpose, EC109 cells in log phase were added to 96-well plates at a density of 1×10^5^ cells/ml (200 µl per well) and then treated as following: FBS-free DMEM for the control, 400 μg/ml CA for low-dose group, 800 μg/ml for middle-dose group and 1600 μg/ml for high-dose group. The cells were treated for 24, 48, 72 or 96 hours. Following administration with different CA concentrations for different durations, 20 ml of MTT solution [5 mg/ml in phosphate buffered saline (PBS)] was added to each well and further incubated for 4 hours at 37˚C. Then, the MTT solution was removed and replaced with 150 µl of dimethyl sulfoxide (DMSO). The plate was further incubated for 5 minutes at room temperature. Optical density (OD) was determined with a Thermo Scientific Microplate Reader (Shanghai, China) at a wavelength of 570 nm. For each experimental condition, five parallel wells were assigned to each group. Experiments were performed three times. 

### Morphological assessment of apoptosis

Apoptotic morphological changes in EC09 cells were detected by Hoechst 33258 staining. In brief, after treatment with different CA concentrations (0, 400, 800 or 1600 µg/ml) for 48 hours, the cells in each group were collected and plated onto the glass slides, followed by fixation in 4% formaldehyde at room temperature. After 20 minutes, fixative reagent was removed, and the cells were washed twice with PBS, stained with 10 μg/ml Hoechst 33258 (Beyotime, China) for 20 minutes and then washed twice with PBS. Nuclear morphological changes in the cells were observed under an Olympus fluorescence microscope (Tokyo, Japan) at 461 nm of emission. 

### Analyses of apoptotic cells by flow cytometry

Annexin V-FITC antibody immunofluorescence combined with propodium iodide (PI)/DNA binding was used to perform a fluorescent analysis of apoptosis. Following incubation with different concentrations of CA for 48 hours, the cells were harvested, centrifuged at 1000 rpm for 5 minutes, and washed two times with pre-cooled PBS buffer. Next, according to the instruction of the Annexin V-FITC apoptosis detection Kit (Beyotime, China), 1×10^5^ cells were collected and incubated with Annexin V-FITC and PI in the provided binding buffer for 30 minutes in dark at 4˚C. They were then subjected to flow cytometer analysis at 488 nm of emission. 

### Lactate dehydrogenase assay

To assess the effect of different concentrations of CA on cell permeability, LDH activity in the medium was measured using LDH assay kit (Nanjing, China) according to the manufacturer’s instruction. Briefly, the cell media was collected after treatment and shortly sonicated. Absorbance value (or OD) was measured by Thermo Scientific Microplate Reader at a wavelength of 490 nm. LDH activity was calculated according to the formula provided by the instructions. 

### Measurement of mitochondrial membrane potential

Mitochondrial activity was measured with JC1 assay kit (Beyotime Institute of Biotechnology). JC1 dye can selectively enter into mitochondria, form J-aggregates and give red fluorescence at a high mitochondrial membrane potential (MMP); whereas, JC1 stays in the cytoplasm with a green fluorescent monomeric form when MMP is relatively low. The ratio between green and red fluorescences provides an estimate of MMP. Briefly, EC109 cells were treated without or with CA (400, 800, 1600 μg/ml) for 48 hour. 200× JC1 stock solution was diluted into 5× JC1 staining buffer which then was added into the cells in suspension and incubated at 37˚C for 20 minutes in dark. Subsequently, the cells were washed twice with PBS. Fluorescence was measured by a flow cytometer using FL1 and FL2 channels (Gallios, Beckman Coulter Inc., USA) and images were taken using a confocal microscope (Zeiss, Germany). 

### Statistical analysis

SPSS 13.0 (Chicago, USA) was used for the statistical analyses. The data are represented as means ± SD. Comparison between multiple groups was performed by one-way analysis of variance (ONE-WAY ANOVA), followed by LSD test. A P<0.05 was considered significant. 

## Results

### Effects of citric acid on EC109 cell proliferation and viability

The effects of CA on EC109 cell proliferation and viability were measured using MTT assay whose measurement is represented as absorbance value. It is well-known that value of OD positively correlates with both cell proliferation rate and survived cell numbers. Thus, as shown in Table 1, proliferation of the EC109 cells was inhibited by this acid in a timeand dose-dependent manner. Particularly, 96 hours after treatment with 400 μg/ml citrate, cell proliferation rate was approximately 52% reduced in comparison with control. At high concentration (1600 µg/ml), CA exhibited the most significant inhibitory effect on cell proliferation rate which was reduced to 31%. Statistical analysis showed that cell viability was significantly decreased (P<0.05) after treatment with both 800 and 1600 µg/ml CA. 

### Effects of citric acid on morphological changes of EC109 cells

**Fig.1:**
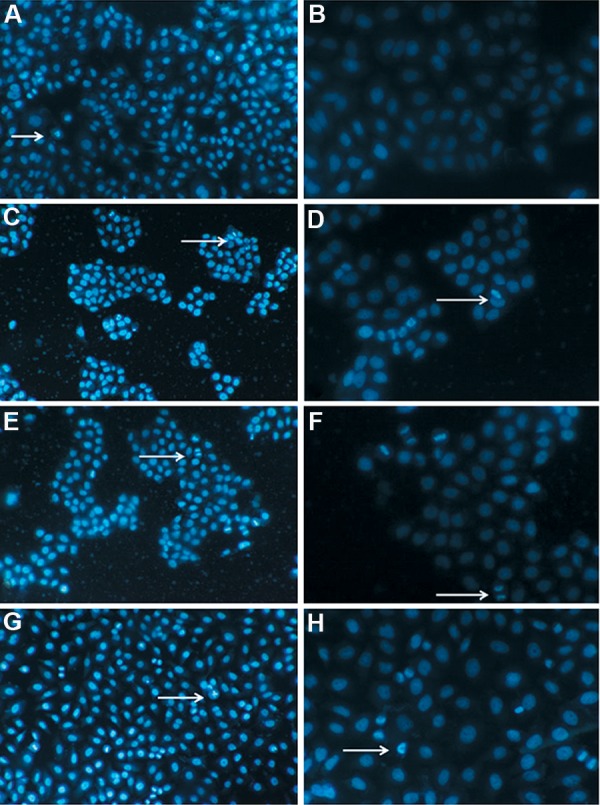
Apoptotic morphological changes in citric acid (CA)-treated EC109 cells. The cells were incubated with 0, 400, 800 and 1600 µg/ml CA for 48 hours. After treatment, apoptotic cells were detected by Hoechst 33258 staining and examined by fluorescence microscope. The representative images of four independent groups are displayed. A., B. Control group, C., D. Low-dose CA treated group (400 μg/ml), E., F. Middle-dose CA treated group (800 μg/ml), G. and H. High-dose CA treated group (1600 μg/ml). Magnification of the left and right images was ×100 and ×400 respectively. Arrows illustrates the representative apoptotic feature of EC109 cell after CA treatment.

Morphological changes of the cells after induction with different doses of CA were observed by Hoechst 33258 staining under fluorescence microscope. As demonstrated in Figure 1, a large proportion of cells in control group grew well and showed intact cell membrane and larger nucleus, while almost no apoptotic nuclei was observed (Fig.1A,B). In contrast, CA-treated groups exhibited typical apoptotic features, including dense fluorescence emission from nuclei and appearance of apoptotic bodies around karyotheca margin. In addition, elevation of CA level resulted in increase in apoptotic cell numbers in a dose-dependent manner (Fig.1C-H). 

**Table 1:** Absorbance value (OD) representing growth changes of the EC109 cells treated with different concentrations of citric acid (CA) for 24, 48, 72 and 96 hours


Groups	Administration time (hours)			
	24	48	72	96

Control (0 µg/ml)	0.964 ± 0.020^a^	0.901 ± 0.074^a^	0.812 ± 0.053^a^	0.759 ± 0.075^a^
Low dosage (400 µg/ml)	0.954 ± 0.010^ab^	0.817 ± 0.010^ab^	0.731 ± 0.090^b^	0.361 ± 0.040^b^
Middle dosage (800 µg/ml)	0.940 ± 0.020^bc^	0.778 ± 0.031^b^	0.681 ± 0.040^cb^	0.331 ± 0.090^bc^
High dosage (1600 µg/ml)	0.927 ± 0.050^c^	0.657 ± 0.08^bc^	0.651 ± 0.020^dc^	0.311 ± 0.030^bd^


Different small letters within the same column mean significantly different (P<0.05); the same letters represent no significant difference (P>0.05).

### Flow cytometric analysis of citric acid-induced EC109 cell apoptosis 

**Fig.2:**
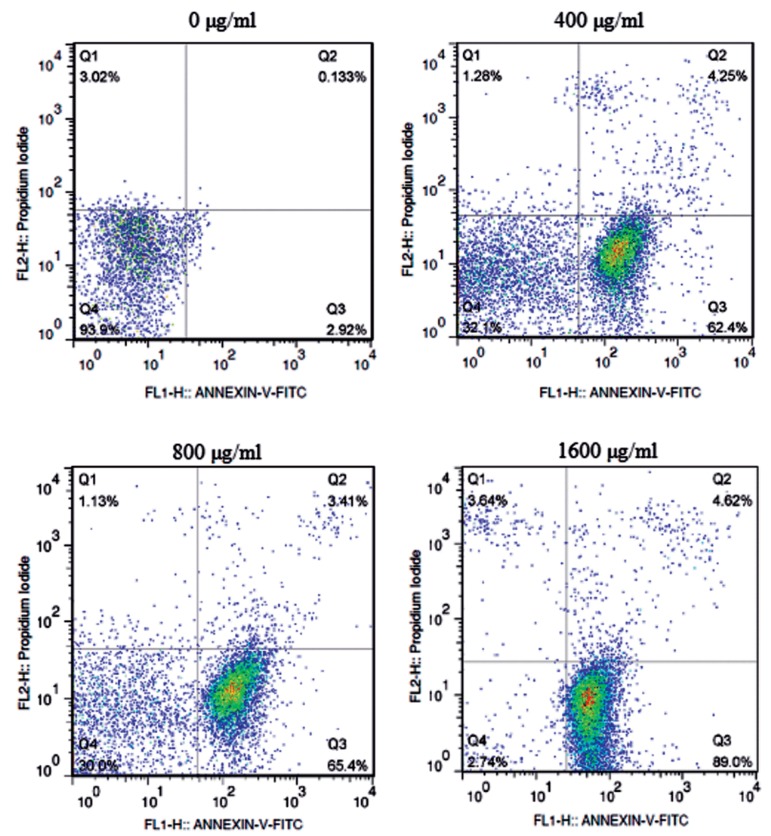
Effects of citrate treatment on EC109 cell apoptosis detected by flow cytometer. The cells, treated with 0, 400, 800 or 1600 µg/ml CA for 48 hours, were evaluated using an Annexin V-FITC/PI staining kit. Cell distribution was analyzed with Annexin V/FITC and PI uptake. FITC and PI fluorescence were measured by flow cytometer with FL-1 and FL-2 filters, respectively. Lower-left quadrant (Q3): living cells (Annexin V-/PI); lower-right quadrant (Q4): early apoptotic/primary apoptotic cells (Annexin V+/PI); upper-left quadrant (Q1): necrotic cells (Annexin V-/PI+); upper-right quadrant (Q2): late apoptotic/secondary apoptotic cells (Annexin V+/PI+). Numbers in the respective quadrant profiles indicate the percentage of the cells presented in this area. CA; Citric acid and PI; Propodium iodide.

To further explore the apoptotic-inducing effect of CA on EC109 cells, in this study, after treatment with CA, the cells were stained with Annexin V-FITC and PI, and then analyzed by flow cytometer. The results demonstrated that CA could dose-dependently induce apoptosis in EC109 cells (Fig.2). When EC109 cells were treated with different concentrations (400, 800, 1600 µg/ml) of CA for 48 hours, percentage of necrotic cells almost did not change or even decreased, whereas the percentage of early apoptotic cells were increased to 62.4, 65.4 and 89.0% respectively, and in the case of late apoptotic cells they were increased to 4.25, 3.41 and 4.62; therefore, total apoptotic cells were increased to 66.65, 68.81 and 93.62%, respectively. These data showed that the number of early apoptotic cells were remarkably increased (P<0.01) along with the increasing level of CA, while increase in late apoptotic cells was more modest. 

### Effect of citric acid on cell permeability 

To test the effect of CA on cellular permeability, release of LDH from the cells into the medium was measured, as an indicator of early cell apoptosis. The result is shown in Table 2, incubation with either 400, 800 or 1600 µg/ml CA for 48 hours caused a significant increase (P<0.01) in LDH release by 1.67-fold, 2.79fold and 3.16-fold, respectively. At the same time, statistical analysis showed an extremely significant difference (P<0.01) in LDH activity in the culture medium between the CA-treated groups. This indicated that treatment with both low-dose and high-dose CA could result in the significant change in cell permeability. 

### Mitochondria membrane potential determination 

**Fig.3:**
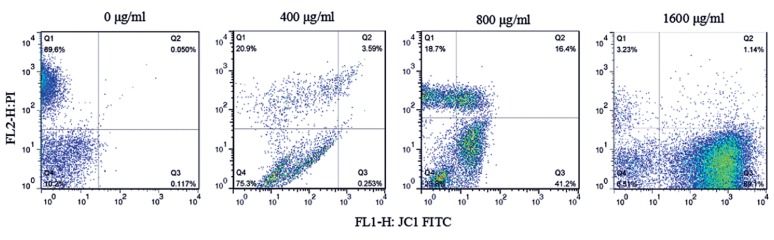
CA-induced mitochondrial depolarization in EC109 cells. Cells were treated with or without CA for 48 hours at the indicated concentrations. Changes in MMP were measured by flow cytometer, using JC1. Q1 (FL1^+^, FL2^-^): JC1 aggregates in healthy mitochondria with high MMP. Q3 (FL1^-^, FL2^+^): JC1 remains as the monomers in the cytoplasm of cells with low MMP. CA; Citric acid and MMP; Mitochondrial membrane potential.

Disruption of mitochondrial integrity is one of the early events leading to apoptosis. To assess whether CA affects mitochondria function, changes in mitochondrial membrane potential were monitored by mitochondria fluorescent dye JC-1. As shown in Figure 3, compared to the control group, treatment with CA for 48 hours significantly reduced the percentage of red fluorescent cells (Q1: JC1-aggregate) from 89.6 to 20.9%, 18.7 and 3.23% at 400, 800 and 1600 μg/ml CA, respectively. Meanwhile, the percentage of green fluorescent cells (Q3: monomer form of JC-1) were increased up to about 0.25, 41 and 89%, suggesting that, overall, CA caused significant decrease of MMP in a dose-dependent manner.

**Table 2:** Effect of different concentrations of CA on LDH activity in the culture supernatant of EC109 cells


Groups	Sample sizes (n)	LDH activity (U/L)

Control(0µg/ml)	5	83.423 ± 11.149^A^
Low dosage (400µg/ml)	5	139.679 ± 12.578^B^
Middle dosage (800µg/ml)	5	232.353 ± 20.187^C^
High dosage (1600µg/ml)	5	263.369 ± 13.845^D^


Different capital letters within the same column indicate very significantly different (P<0.01). LDH; Lactate dehydrogenase and CA; Citric acid.

## Discussion

CA belongs to a kind of organic acid distributing widely in varieties of plants, and presented in high concentration in lemon juice. This organic acid has an important role in animal metabolism. CA appears to intermediate relative cycle, as one of the most important metabolic pathways, in eukaryotic cells. In mammals CA level can directly regulate several metabolic pathways and increase calcium uptake from foods (17). In addition, as a potential inhibitor of PFK, increasingly evidences have indicated that a high level of citrate could inhibit the glycolytic pathway required for cancer growth (13,18). However, whether citrate could induce apoptosis in EC cells remains yet unclear. 

The results from the MTT assay indicated that CA inhibited proliferation of EC109 cells in a timeand dose-dependent manner. These observations were consistent with previous reports, indicating that CA induced obvious cytostaticity against BGC-823 and SGC-7901 cells (18), although EC109 cells were less sensitive to citrate than two indicated cell lines. Findings obtained from the MTT assay in our study showed that administration with high concentration of CA (1600 µg/ml) for 96 hours caused a nearly 60% decrease in cell viability compared to the control group. 

To further explore whether inhibition of EC109 cells growth by CA was associated with apoptotic mechanism, nuclear morphological changes were detected by Hoechst 33258 fluorescent staining. Results showed that, independent to concentration, the CA-treated cells exhibited characteristic features of apoptosis, such as chromatin condensation, nuclear fragmentation and appearance of apoptotic bodies as well. In addition, induction of apoptosis was confirmed by flow-cytometer after Annexin V-FITC and PI staining in this study, whereby CA-administration for 48 hours caused a dose-dependent increase in the percentage of apoptotic cells. CA has also been reported to induce apoptosis in BGC-823 and SGC-7901 cells after exposure to citrate (18). These results confirmed that CA exposure could induce the apoptotic death of EC109 cells. 

It has been shown that one of the features of cell apoptosis is increase in cell membrane permeability. LDH is an intracellular enzyme released outside of the cells when cell membrane is disintegrated. Therefore, apoptotic cell death can be estimated by measuring the level of LDH releasing in medium from cytoplasm. In our study, data analysis showed that LDH levels in the medium from all of the CA-treated groups were significantly different from control, which was in agreement with the results obtained from MTT assay, as described above. Statistical analysis also showed a very significant difference in LDH activity between two CA-treated groups. This suggests that CA remarkably impacted the leakage rate of LDH, and LDH activity was elevated gradually as the concentration of CA was increased, also implying an increase in cell membrane permeability caused by CA administration. 

Mitochondrion is an important intracellular organelle, with a crucial function in ATP generation, which is reliant on the stability of MMP. Lines of evidence have indicated that this organelle plays a critical role in apoptosis regulation (19,20). Loss of MMP was thought to be associated with mitochondrial dysfunction, leading to apoptosis (21). Additionally, loss of MMP may lead to the rupture of outer membrane or opening specific channels which are responsible for release of the apoptogenic molecules into cytosol, and finally triggering mitochondria-dependent cell apoptotic pathway (22). In the present study, EC109 cells exposed to various concentrations of CA (400, 800, 1600 μg/ml), resulted in dissipation of MMP in the concentration-dependent manner. Our study suggested that mitochondrial damage might be related to EC109 cell apoptosis induced by CA. 

All of the above results, taken together, suggested that CA exhibited an inhibitory effect on cell growth by inducing apoptosis. Similarly, the impacts of CA on tumor cell growth were reported by Zhang et al. (23) and Bucay (24) who observed an anti-tumor effect after treatment with citrate. Whereas, the mechanism(s) underlying cell apoptosis, caused by citrate treatment, still remain to be elucidated. Nonetheless, it is proposed that induction effects of EC109 cell apoptosis by CA may be associated with inhibition of glycolysis process in tumor cells. CA citrate level in the cells is a key factor in the regulation of energy metabolism. As an intermediate in the TCA cycle, citrate induces a negative feedback on glycolysis through inhibiting PFK or PK enzyme activity. For instance, Liu et al. (15) showed that adding sodium citrate resulted in remarkable decrease in the activities of several key enzymes including PFK and PK, followed by reduction of glycolytic flux. This is likely the case in tumor cells, meaning that administration of excessive citrate could inhibit activities of these enzymes sufficiently to arrest glycolysis, which would cause severe energy depletion in cancer cells, leading to cell growth arrest or even apoptosis. Interestingly, Halabe Bucay (25) also suggested that administration of citrate indeed slowed tumor cell growth, which was similar to the findings of our study. However, the exact mechanism linking glycolysis to apoptotic pathways remains unclear. 

## Conclusion

Present study determined that CA induced apoptosis in EC109 cells in a timeand concentrationdependent manner. In parallel, our results showed that the exposure of CA to EC109 cells raised LDH activity in the supernatant medium and disrupted mitochondrial membrane potential. Moreover, CA treatment induced a strong apoptotic effect on EC109 cells, such as nuclear condensation fragmentation, and the occurrence of apoptotic body as well. Therefore, according to the present data and the findings of other studies, CA induces growth inhibition and apoptosis in EC cells possibly by preventing glycolytic pathway, through inhibiting the activity of glycolytic enzymes including PFK, PK or LDH. Thus, it abolishes generation of necessary energy for tumor cell proliferation, finally leading to tumor cell apoptosis which is reflected by increased LDH leakage and reduced mitochondrial membrane potential. 

## References

[1] Aurello P, D'Angelo F, Rossi S, Bellagamba R, Cicchini C, Nigri G (2007). Classification of lymph node metastases from gastric cancer: comparison between N-site and Nnumber systems.Our experience and review of the literature. Am Surg.

[2] Wang JB, Fan JH, Liang H, Li J, Xiao HJ, Wei WQ (2012). Attributable causes of esophageal cancer incidence and mortality in China. PLoS One.

[3] Icarda P, Poulaina L, Linceta H (2012). Understanding the central role of citrate in the metabolism of cancer cells. Biochim Biophys Acta.

[4] Kruspig B, Nilchian A, Orrenius S, Zhivotovsky B, Gogvadze V (2012). Citrate kills tumor cells through activation of apical caspases. Cell Mol Life Sci.

[5] Angumeena AR, Venkappayya D (2013). An overview of citric acid production. LWT-Food Sci Technol.

[6] Dhillon GS, Brar SK, Verma M, Tyagi RD (2011). Recent advances in citric acid bio-production and Recovery. Food Bioprocess Tech.

[7] Wang Z, Jeon HY, Rigo F, Bennett CF, Krainer AR (2012). Manipulation of PK-M mutually exclusive alternative splicing by antisense oligonucleotides. Open Biol.

[8] Cortés-Cros M, Hemmerlin C, Ferretti S, Zhang J, Gounarides JS, Yin H (2013). M2 isoform of pyruvate kinase is dispensable for tumor maintenance and growth. Proc Natl Acad Sci USA.

[9] Huang L, Yu Z, Zhang T, Zhao X, Huang G (2014). HSP40 interacts with pyruvate kinase M2 and regulates glycolysis and cell proliferation in tumor cells. PLoS One.

[10] Fan J, Hitosugi T, Chung TW, Xie J, Ge Q, Gu TL (2011). Tyrosine phosphorylation of lactate dehydrogenase A is important for NADH/NAD(+) redox homeostasis in cancer cells. Mol Cell Biol.

[11] Zhao Y, Butler EB, Tan M (2013). Targeting cellular metabolism to improve cancer therapeutics. Cell Death Dis.

[12] Jenkins CM, Yang J, Sims HF, Gross RW (2011). Reversible high affinity inhibition of phosphofructokinase-1 by acyl-CoA: a mechanism integrating glycolytic flux with lipid metabolism. J Biol Chem.

[13] Hatzivassiliou G, Zhao F, Bauer DE, Andreadis C, Shaw AN, Dhanak D (2005). ATP citrate lyase inhibition can suppress tumor cell growth. Cancer Cell.

[14] Li JJ, Wang H, Tino JA, Robl JA, Herpin TF, Lawrence RM (2007). 2-hydroxy-N-arylbenzenesulfonamides as ATPcitrate lyase inhibitors. Bioorg Med Chem Lett.

[15] Liu X, Chen S, Chu J, Zhuang Y, Zhang S (2004). Effect of sodium citrate on the growth metabolism and inosine accumulation by Bacillus subtilis. Acta Microbiologica Sinica.

[16] Gu SZ, Zhao BC (1991). The inhibition of citrate on lactate dehydrogenase in rabbit heart muscle. DaLian YiXue XueBao.

[17] Gautier-Luneau I, Bertet P, Jeunet A, Serratrice G, Pierre JL (2007). Iron-citrate complexes and free radicals generation: is citric acid an innocent additive in foods and drinks?. Biometals.

[18] Lu Y, Zhang X, Zhang H, Lan J, Huang G, Varin E (2011). Citrate induces apoptotic cell death: a promising way to treat gastric carcinoma?. Anticancer Res.

[19] Indran IR, Tufo G, Pervaiz S, Brenner C (2011). Recent advances in apoptosis, mitochondria and drug resistance in cancer cells. Biochim Biophys Acta.

[20] Giorgi C, Wieckowski MR, Pandolfi PP, Pinton P (2011). Mitochondria associated membranes (MAMs) as critical hubs for apoptosis. Commun Integr Biol.

[21] Kroemer G, Galluzzi L, Brenner C (2007). Mitochondrial membrane permeabilization in cell death. Physiol Rev.

[22] Cregan SP, Dawson VL, Slack RS (2004). Role of AIF in caspase-dependent and caspase-independent cell death. Oncogene.

[23] Zhang X, Varin E, Allouche S, Lu Y, Poulain L, Icard P (2009). Effect of citrate on malignant pleural mesothelioma cells: a synergistic effect with cisplatin. Anticancer Res.

[24] Bucay AH (2011). Clinical report: a patient with primary peritoneal mesothelioma that has improved after taking citric acid orally. Clin Res Hepatol Gastroenterol.

[25] Halabe Bucay A (2009). Hypothesis proved.citric acid (citrate) does improve cancer: a case of a patient suffering from medullary thyroid cancer. Med Hypotheses.

